# Developing a novel methodology to evaluate the content validity of two sensory reactivity assessments adapted for South Africa

**DOI:** 10.1177/03080226251348042

**Published:** 2025-07-21

**Authors:** Ann Frances Watkyns, Lizahn Gracia Cloete, Linda Diane Parham

**Affiliations:** 1 Division of Occupational Therapy, Department of Medicine and Health Sciences, Stellenbosch University, Stellenbosch, South Africa; 2 Paediatrics Department of the School of Medicine, University of New Mexico, Albuquerque, NM, USA

**Keywords:** Sensory modulation, test adaptation, child, psychometrics

## Abstract

**Introduction:**

When a standardised assessment is used in a new context or with a population that differs from the original assessment’s normative sample, content validity should be re-evaluated. This study reports on the methodology developed for evaluating content validity of two sensory reactivity assessments that were adapted for use in the Western Cape province, South Africa.

**Method:**

A sequential explanatory mixed-method approach was used. The first part of the evaluation of content validity employed a qualitative methodology with cognitive interviewing of participants after administration of the adapted sensory reactivity assessments in a feasibility study. The second part of the evaluation involved a quantitative methodology to rate item and subscale content validity by an expert group of occupational therapists.

**Results:**

Most of the measures of content validity evaluated indicated sufficient content validity. Where there were insufficient content validity ratings, adaptations were made to increase the content validity of the assessments.

**Conclusion:**

A novel methodology for evaluating the content validity of an assessment was developed and used to evaluate the content validity of two sensory reactivity assessments that had been adapted for use in the Western Cape province. Thresholds were set for determining sufficient content validity of both the qualitative and quantitative data.

## Introduction

Content validity is concerned with whether the content of the test evaluates the construct being measured (De Vet et al., 2011). Some experts in measurement theory consider content validity the most important psychometric property (Terwee et al., 2018b). The COnsensus-based Standards for the selection of health status Measurement INstruments (COSMIN) group provides some guidelines for evaluating content validity and recommend that if content validity is insufficient, no further psychometric properties should be evaluated (Terwee et al., 2018b). Some methodological limitations were identified in the literature.

Content validity is not an inherent characteristic of an assessment, as findings only apply to the context and population where the content validity was evaluated (De Vet et al., 2011). When an assessment is used in a new context, or with a new population, content validity needs to be re-evaluated to ensure appropriateness in the new context (De Vet et al., 2011).

This study formed the third and final stages of a larger research study. In the first stage, a paediatric sensory reactivity (SR) assessment suitable for adaptation was identified (Watkyns et al., 2023). The second stage described the adaptation of the SR assessment so that it would be contextually appropriate for the Western Cape province, South Africa (Watkyns et al., 2024). The SR assessment included some items from the Sensory Processing Measure 2nd edition (SPM-2) caregiver questionnaires for reschool (ages 2–5 years) and Child (ages 5–12 years), and seven tests evaluating SR from the Evaluation in Ayres Sensory Integration (EASI), a performance-based assessment. This article reports on stage three, where the aim was to evaluate content validity of the adapted assessment. The research question was: Which method can comprehensively evaluate the content validity of two adapted SR assessments?

## Literature review

SR difficulties are characterised by problems regulating responses to sensory stimuli that most people would easily adapt to. This impacts quality of life, functional skills and emotional and mental well-being in both children and adults (Parham et al., 2021).

The COSMIN researchers consider content validity to comprise several aspects. These include feedback from patients on the relevance, comprehensibility and comprehensiveness of the assessment; feedback from professionals on the relevance and comprehensiveness of the assessment; and a rating by reviewers of the content validity of the test items (Terwee et al., 2018b). *Relevance* refers to whether the test items reflect the construct being measured (Terwee et al., 2018b). In this study, the construct was SR. *Comprehensibility* focuses on whether the patient understands the test instructions and test items as intended by the tester (Terwee et al., 2018b). Information on comprehensibility should only be elicited from patients, not professionals, as professionals cannot give an objective opinion due to their familiarity with the terminology used in the field (Terwee et al., 2018b). This is contrary to many SR adaptation studies in the literature, which elicited feedback solely or primarily from professionals (Hadgu and Zeleke, 2017; Lai et al., 2011). *Comprehensiveness* evaluates whether all key aspects of the construct had been included in the assessment (Terwee et al., 2018b).

Acceptability of the assessment was not identified by the COSMIN group as an aspect of content validity (Terwee et al., 2018b), but was considered by other authors to be an important factor (International Test Commission 2017; Smart, 2006). Acceptability denotes the suitability of an assessment for participating communities or individuals. Test items may be considered unacceptable due to discomfort (Smart, 2006), contextual inappropriateness (International Test Commission 2017) or negative societal or ethical implications (Smart, 2006). The authors of this study considered it important that the test items were acceptable to both patients and professionals, as contextual appropriateness was a significant consideration when adapting the assessment for the South African context.

Semi-structured cognitive interviews were used in some studies to identify possible barriers experienced by participants when participating in an SR assessment (Gandara-Gafo et al., 2021; Román-Oyola and Reynolds, 2010; Shahbazi et al., 2021).

Other studies used informal, unstructured interviews or questionnaires to obtain feedback (Lai et al., 2011; Su and Parham, 2002). Guidelines for thresholds to determine sufficient content validity were reported only by the developers of the cognitive interviewing methodology (Willis, 2005) and by the authors of two test adaptation studies (Gandara-Gafo et al., 2021; Román-Oyola and Reynolds, 2010). One test adaptation study stipulated that if three or more of eight participants noted comprehension difficulties, the item was deemed to lack sufficient content validity and needed adaptation (Román-Oyola and Reynolds, 2010). The second test adaptation study (Gandara-Gafo et al., 2021) and the cognitive interview developers (Willis, 2005) recommended that if two or more participants identified challenges with the language used, irrespective of the total number of participants, that test item should be revised.

A further aspect of content validity is the rating of individual test items and subscales. Test development studies on a limited number of SR assessments rated individual test items (Sutthachai et al., 2022). However, this methodology was only used in one SR test adaptation study (Shahbazi et al., 2021). There was consensus in the literature that item and subscale rating should be performed by professionals knowledgeable in the field (Rovinelli and Hambleton, 1976; Terwee et al., 2018b; Turner and Carlson, 2003). However, the type of rating scale used, the method of determining the final rating score and how these scores were interpreted, varied across studies.

The COSMIN group uses a four-point rating scale to rate individual items of an instrument (Terwee et al., 2018b). Other studies have used a three-point rating scale (Rovinelli and Hambleton, 1976; Sutthachai et al., 2022; Thorn and Deitz, 1989; Turner and Carlson, 2003). A variety of methods was used in these studies to statistically calculate the final score, such as percentage of agreement, proportion of agreement or index of item-objective congruence (IOC) (Shahbazi et al., 2021; Sutthachai et al., 2022; Terwee et al., 2018a; Thorn and Deitz, 1989; Turner and Carlson, 2003). Minimum IOC scores for sufficient item content validity rating varied between 0.50 and 0.80 (Rovinelli and Hambleton, 1976; Sutthachai et al., 2022; Turner and Carlson, 2003).

The final aspect of item content validity was determination of the percentage of items that represented the construct, out of the total items. This was done in very few studies. The COSMIN recommendation is that a minimum of 85% of test items should reflect the construct, in order for the content validity of the test to be considered acceptable (Terwee et al., 2018a). A similar method was used in the study of the Test of Sensory Functions in Infants (DeGangi et al., 1988), which stated that 75%–85% of the test items should represent the construct, although the exact threshold was not reported.

The components of content validity outlined above were met in very few studies on SR assessments. Three studies on the development of SR assessments for children reported conducting formal content validity studies (Johnson-Ecker and Parham, 2000; Schoen et al., 2008; Sutthachai et al., 2022). However, the content validity studies were limited in scope and did not cover all the aspects outlined above. Several content validity studies of adapted SR assessments provided insufficient information on their research findings (Benjamin et al., 2014; Lai et al., 2011; Román-Oyola and Reynolds, 2010; Shahbazi et al., 2021). Román-Oyola and Reynolds (2010) reported that ‘[based] on the information collected through the cognitive interviews in this preliminary study, it is considered that the [Spanish] Short Sensory Profile demonstrated validity’ (Román-Oyola and Reynolds, 2010: 204), without specifying the criteria that guided this decision. Another test adaptation study in India reported that content validity was adequate, although the authors did not report how this was analysed or how this conclusion was reached (Benjamin et al., 2014). The following statement in this article, ‘The [. . .] content validity [. . .] has long been established by consensus among clinicians during the development of the Sensory Profile . . . [and] is a well-established measure for sensory processing in other countries, cultures and contexts’ (Benjamin et al., 2014: S185), indicates a misunderstanding of the principle that content validity applies only in the context where it originated, and cannot be assumed to have the same value in a different context (De Vet et al., 2011).

Only one study, a test adaptation study, reported obtaining feedback from children (Gandara-Gafo et al., 2021). Cognitive interviews were conducted with eight children whose ages ranged from 3 to 6 years. The interviews involved reading the test instructions to the children and then asking them to repeat the instructions in their own words (Gandara-Gafo et al., 2021).

There are significant language, socio-economic, cultural and educational differences between South Africa and the United States, where the EASI and the SPM-2 were developed. Content validity may therefore not have the same value in South Africa as in the United States and needs to be re-evaluated. This article reports on the comprehensive methodology developed to evaluate the content validity of these adapted SR assessments.

## Methodology

Evaluation of the content validity of the adapted SR assessment employed a sequential explanatory mixed-method approach. The first part of the evaluation used a qualitative methodology that consisted of cognitive interviewing of participants in a feasibility study. The second part of the evaluation involved a quantitative methodology to rate item and subscale content validity by an expert group of occupational therapists. Ethical approval was obtained from Stellenbosch University, HREC Reference No: S21/02/029 (PhD) for 2021 to 2023, to conduct the research. All participants involved in each part of the evaluation were required to sign consent forms in their home language.

The feasibility study was conducted using the 2 adapted SR assessments with 30 children aged 3 to 12 years and their caregivers. The sample size was guided by the literature (Beaton et al., 2007; de Klerk et al., 2020). Stratified random sampling was employed to reflect the population of the Western Cape province in terms of gender, age, rural/urban environments and socio-economic status. The purpose of the feasibility study was to evaluate aspects of the content validity of the adapted assessments. The performance-based component of the assessment was administered by five occupational therapists and the principal investigator (PI) with the children, and their caregivers completed the caregiver questionnaire. After the testing had been completed, cognitive interviews were conducted by the occupational therapists with the caregivers and with children aged 7 years and older, after which the PI conducted cognitive interviews with the occupational therapists. Children younger than 7 years were excluded, maintaining consistency with the ethical guideline that children younger than this were judged to be too young to make a decision regarding informed consent (Human Research Ethics Committee Faculty of Health Sciences University of Cape Town, 2013). A cognitive interview guide was developed by the PI to provide a similar structure to all the interviews, while allowing for individual variations according to the participants’ responses (Terwee et al., 2018b). The interviews aimed to explore the challenges experienced in using the adapted SR assessments.

A second expert panel of four occupational therapists was selected by the PI to conduct item and subscale content validity ratings. These were experts in the field of paediatric sensory integration with a minimum of 10 years’ experience. Most were not familiar with the concept of content validity. Training in content validity was therefore undertaken by the PI to enable them to fulfil the functions required of them. This training involved explaining the concepts of content validity and performing two item-rating exercises. In these exercises, the occupational therapists were required to rate the content validity of test items of the Clinical Observations of Gross Motor Items (South African Institute for Sensory Integration: Clinical Observations Revision Committee, 2003), a paediatric assessment developed for the South African population, and the Short Sensory Profile-2 (Dunn, 2014). Group discussion continued until all occupational therapists understood the process.

### Data collection

All 30 caregivers were requested to provide information on the comprehensibility, acceptability, relevance and comprehensiveness of the caregiver questionnaire. Eighteen children 7 years and older were asked about the comprehensibility and acceptability of the performance-based component. The PI judged these to be appropriate concepts for children in this age group to be able to understand and describe. The PI then interviewed the occupational therapists to elicit information on the acceptability, relevance and comprehensiveness of both the caregiver questionnaire and the performance-based component. The cognitive interview responses were recorded and transcribed verbatim. All test sheets and cognitive interview data were numbered, with no names recorded, to ensure confidentiality.

The second expert panel of four occupational therapists independently rated the item and subscale content validity of the adapted SR assessments according to the three-point rating scale of 1 (definitely measures the construct), −1 (definitely does not measure the construct) and 0 (unsure if measures the construct) (Sutthachai et al., 2022; Thorn and Deitz, 1989; Turner and Carlson, 2003). Independent rating was done to reduce bias from the PI or other occupational therapists, as bias might influence responses if rating decisions were discussed. In addition, no moderation was done by the PI. This increased the validity and reliability of the rating results. A final score for each test item and subscale was calculated by the PI using percentage of agreement (Thorn and Deitz, 1989).

### Data analysis

The data obtained from cognitive interviews with the children, caregivers and occupational therapists constituted the primary qualitative data. Thematic analysis of the primary data was done manually. Inductive analysis was used, with categories and themes allowed to arise organically, rather than being predetermined (de Klerk et al., 2020; Terwee et al., 2018a). This method enhanced the validity of the data analysis and prevented possible bias that might result if themes were preselected by the PI. A list of the identified challenges was drawn up, with each test item related to a specific challenge, along with an explanation of why each challenge presented a problem. The reasons for the challenges were then organised into related categories. The categories were then grouped together thematically. Main themes were broken down into sub-themes according to categories within each main theme. The PI used a percentage of agreement of 75% between the participants to indicate sufficient content validity from the cognitive interview data. This cut-off was based on limited guidelines in the literature (Gandara-Gafo et al., 2021; Román-Oyola and Reynolds, 2010; Willis, 2005) as well as the PI’s clinical experience, which indicated that some atypical responses can occur naturally.

The ratings of the five reviewers (the four occupational therapists and the PI) on each test item and subscale were combined using the percentage of agreement statistic. A cut-off criterion of 80% for the percentage of agreement reflected that 80% of the total number of test items in the subscale represented the construct, indicating sufficient content validity. In this study, the test items in each subscale achieving below the 80% cut-off were analysed by the PI to determine whether the whole subscale or individual items should be removed from the assessment. The decision regarding the removal of items was guided by considerations of the sensory system involved and the item’s importance in Ayres Sensory Integration^®^ theory and practice, as well as its relevance to the construct of SR.

## Results and findings

Post-test cognitive interview feedback from the child and caregiver participants, as well as the occupational therapists, indicated numerous challenges related to the adapted SR assessments. Two themes emerged from the data analysis. Theme One represented challenges related to language. Theme Two represented challenges related to inappropriate or threatening assessment tasks. The identified challenges are discussed below, with selected quotations from participants presented verbatim.

### Cognitive interviews with children

Most children indicated no challenges in doing the performance-based assessment. All children understood the instructions, indicating no difficulties with comprehension or language difficulties in Theme One. Four children identified challenges in Theme Two, inappropriate or threatening assessment tasks, which related to the acceptability of the assessment. These four challenges did not fall below the threshold for sufficient validity. Their comments are reproduced below:

Child 23 (9 years): *That one where you tipped me back on the chair*.
Child 8 (9 years): *Nee . . . die goed wat ek in my mond moet sit was nie lekker nie* [no . . . it wasn’t a nice feeling having the stuff in my mouth].
Child 13 (12 years): *Yes . . . when I had to jump off the step with my eyes closed*.
Child 15 (8 years): *Yes . . . except for putting those things in my mouth and the smells . . . I got a bit nervous with the chair, when I was falling back*.

These findings indicated sufficient content validity for the acceptability and comprehensibility of the assessment. Table 1 shows the counts for these two aspects of content validity.

**Table 1.** Content validity with children.

Content validity category	Children (7 years and older) without concerns (*N* = 18)	
	*n*	%
Comprehensibility	18	100
Comprehensiveness^a^	NA	NA
Relevance^a^	NA	NA
Acceptability	14	78

NA: not applicable; *N*/*n*: number.

a Not evaluated with children.

### Cognitive interviews with caregivers

Many language challenges related to Theme One were experienced with the caregiver questionnaire. The sub-theme of contextually inappropriate language related to acceptability. The threshold indicated sufficient content validity for acceptability. The four items identified by three caregivers as contextually inappropriate are provided below:

Caregiver 26: *We don’t have pets* [SPM-2 South African adaptation (SPM-2 SA) Preschool item 44 – Pats animals with too much force].
Caregiver 26: *We don’t have a vacuum cleaner* [SPM-2 SA Preschool item 11 – Is bothered by ordinary household sounds, such as the vacuum cleaner].
Caregiver 29: *My child doesn’t know this* [SPM-2 SA Preschool item 15 – Is upset by shrill sounds such as whistles or party noisemakers].
Caregiver 25: *We use communal toilets and these are dirty and dangerous and our children don’t want to use them* [SPM-2 SA Preschool item 24 – Seems unaware of the need to use the toilet].

The Theme One sub-theme of language complexity or statements lacking clarity reflected comprehensibility. This sub-theme presented the most challenges, resulting in insufficient content validity for comprehensibility. Ten caregivers needed the test instructions explained more fully. Five out of the 10 caregivers also required translation assistance for the whole questionnaire. Words or phrases frequently not understood included slumps, startles, bothered, distracted, squints, flips, distinguish.

Another aspect of this sub-theme was statements that lacked clarity, although caregivers did understand the words used in the statement. This was reflected in three test items, each highlighted by one caregiver. These were SPM-2 SA Preschool item 8 – Is distracted by visible objects or people (the caregiver considered this a normal behaviour); SPM-2 SA Child item 4 – Has trouble following moving objects with their eyes (the caregiver said she would not notice this behaviour in the child); SPM-2 SA Child item 26 – Has an unusually high tolerance for pain, showing little or no distress at minor injuries (Caregiver 15: *She has a very low tolerance. What do I say . . . I don’t know which option to choose*). There was no option provided in the statement for a child who had low pain tolerance.

All caregivers reported that the test items were relevant and that the test was comprehensive, with sufficient content validity for these two aspects. See Table 2 for counts of content validity with the caregivers, with a further breakdown expanding on the types of comprehensibility challenges that occurred.

**Table 2.** Content validity with caregivers.

Content validity category	Caregivers without concerns (*N* = 30)	
	*n*	%
Comprehensibility	14	47
Comprehensiveness	30	100
Relevance	30	100
Acceptability	26	87

Comprehensibility	Caregivers without concerns (*N* = 30)	
	*n*	%
Major translation assistance	25	83
Understanding specific word or phrase	18	60
Item vagueness	27	90

*N*/*n*: number.

### Cognitive interviews with occupational therapists

The occupational therapists all found that the assessments were relevant to the construct of SR, and comprehensive, thus demonstrating sufficient content validity for these aspects. Challenges that were identified related to the same two themes reported on by caregivers, namely Theme One: Contextually inappropriate language, and Theme Two: Challenges related to inappropriate or threatening assessment tasks. Both of these categories of challenges related to acceptability.

Theme One (contextually inappropriate language) contained the same four test items with contextually inappropriate words, as noted by caregivers, namely a vacuum cleaner, toileting, pets and party noisemakers. Theme Two challenges were related to inappropriate or threatening assessment tasks. One concern related to the level of difficulty for children in matching textures in the Tactile Perception: Shapes test. Two occupational therapists reported that none of the children they tested could respond correctly to any of these test items. A second challenge related to the SR test item that involved tipping a chair back. The third challenge was identified in the Tactile Perception: Oral test regarding a possible risk of spreading tuberculosis. The assessment was therefore rated as having insufficient content validity for acceptability. See Table 3 for counts of the three aspects of content validity evaluated by the occupational therapists, with further analysis expanding on the acceptability challenges.

**Table 3.** Content validity with occupational therapists.

Content validity category	Occupational therapists without concerns (*N* = 4)	
	*n*	%
Comprehensibility^a^	NA	NA
Comprehensiveness	3	75
Relevance	4	100
Acceptability	1	25

Acceptability	Occupational therapists without concerns (*N* = 4)	
	*n*	%
Vacuum cleaner	2	50
Pets	3	75
Toileting	3	75
Whistles and party noise makers	3	75
Too difficult for children	2	50
Oral	3	75
Tipping chair backwards	3	75

NA: not applicable; *N*/*n*: number.

a Not evaluated with occupational therapists.

### Item content validity ratings

All of the items and subscales that were derived from the EASI, the performance-based component of the SR assessment, demonstrated 100% agreement. Varying item content validity ratings were obtained for the SPM-2 SA Preschool and Child caregiver questionnaires, and these are summarised for the SPM-2 SA Child questionnaire, according to the relevant sensory system subscales. The vision subscale demonstrated insufficient item content validity as only five items were rated to measure the construct of SR, less than the 80% cut-off for sufficient item content validity. The hearing, touch, and taste and smell subscales demonstrated sufficient item content validity. However, the body awareness subscale, which reflects integration of proprioceptive input, demonstrated insufficient item content validity, with only two items rated as measuring the construct. The balance and motion subscale, which reflects vestibular sensory integration, also had insufficient item content validity as only three items were rated to measure the construct. A summary of the overall item and subscale content validity ratings of the adapted SR assessment, with the percentage of agreement, is reproduced in Table 4. Only the results for the SPM-2 SA Child questionnaire are presented.

**Table 4.** Count of test items by percentage of agreement, test and subscales.

Test	Subscales	Percentage of agreement						Total number of test items
		0%	20%	40%	60%	80%	100%	
	Hyperreactivity only	0	0	0	0	0	19	19
EASI	Hyper-reactivity and Hypo-reactivity combined	0	0	0	0	0	9	9
SPM-2 SA Child	Vision	1	2	2	0	1	4	10
SPM-2 SA Child	Hearing	0	0	0	0	1	9	10
SPM-2 SA Child	Touch	0	1	0	0	0	9	10
SPM-2 SA Child	Taste and smell	0	0	1	0	2	7	10
SPM-2 SA Child	Body awareness	1	2	2	3	2	0	10
SPM-2 SA Child	Balance and motion	3	2	1	1	0	3	10
		5	7	6	4	6	60	88

EASI: Evaluation in Ayres Sensory Integration; SPM-2: Sensory Processing Measure 2nd edition.

## Discussion

Content validity reflects whether the content of the test evaluates the construct that it purports to measure (Kielhofner, 2006; Terwee et al., 2018b). Content validity of the two adapted SR assessments was therefore the first measurement property to be evaluated in this study. A comprehensive methodology was developed for this study to evaluate content validity.

### Cognitive interviews

Information on relevance, comprehensiveness, comprehensibility and acceptability was collected using cognitive interviews. The sufficient content validity rating for comprehensibility by children in the performance-based component, reflected the administration of this part of the assessment. This administration was designed with simple and brief verbal instructions that were not highly standardised. This allowed the tester to make changes when the child experienced difficulties in understanding the instructions (Mailloux et al., 2020). The test administration also allowed for alternate or additional verbal instructions as well as informal translations into the children’s home languages (Mailloux et al., 2020). In addition, trial items and the use of demonstrations by the tester were incorporated into the test administration, aiding the children’s understanding. One SR movement item initially identified as unacceptable (child’s chair tipped back suddenly) was revised to include the statement by the occupational therapist ‘I won’t do that again’ after completion of the item. This was aimed at reducing any anxiety the child may have experienced, by reassuring them that the item would not be repeated.

The caregiver cognitive interview data rated comprehensiveness, relevance and acceptability as sufficient. However, the contextual appropriateness, and thus acceptability of this assessment for the South African context was important, and has been emphasised in the literature (Hadgu and Zeleke, 2017; International Test Commission, 2017; Lai et al., 2011). Therefore, three objects that caregivers said their children were not familiar with, namely pets, vacuum cleaner and party noisemakers, were either removed or replaced with similar but appropriate items to render them acceptable. A precedent has been set for this in the literature where items were removed or changed to reflect the culture for which the assessment was being adapted (Hadgu and Zeleke, 2017; Lai et al., 2011). An additional item of the SPM-2 SA Preschool questionnaire, item 24 ‘seems unaware of the need to use the toilet’ was considered contextually inappropriate by one of the caregivers, as they did not have a toilet inside their home and had to use communal toilets. These were dirty and dangerous, and their children were reluctant to use them and may suppress the need to use the toilet. Communal toilets or outside pit toilets are features of many low socio-economic communities in South Africa. It is therefore likely that many children in South Africa who may be assessed with this questionnaire in future will be using these facilities. While the SPM-2 test developers interpreted this test item as most likely to reflect sensory hypo-reactivity (Parham et al., 2021), it did not take into account this feature of the South African context. This item was therefore removed from the questionnaire. However, toileting challenges may be present in children with SR difficulties (Parham et al., 2021), and exclusion of this item without replacement with a similar toileting item is problematic. It is therefore recommended that therapists assess the child’s toileting function by using other assessment procedures such as informal interviewing of the caregiver or staff in the preschool educational setting to elicit accurate information.

The comprehensibility of the caregiver questionnaire was rated by caregivers as insufficient. Language challenges were reported primarily where the caregivers’ home language was not English. When English was the second or third language, caregivers may have been at a disadvantage when completing the questionnaire, affecting the accuracy of their responses. Similar language challenges, where English was not the caregiver’s home language, have been reported in the literature (Hadgu and Zeleke, 2017; Shahbazi et al., 2021). Challenges in comprehension when using the Likert scale were addressed by replacing the abbreviations as descriptors of the Likert scale by the words printed out in full, with ‘A’ replaced by ‘Always’, and so on, to reduce confusion. Similar difficulties with understanding the Likert scale descriptors was noted in a study with low socio-economic participants from the Western Cape province when completing a questionnaire (de Klerk et al., 2020). These authors replaced the numbers with words as scale descriptors (de Klerk et al., 2020).

One occupational therapist identified concerns regarding possible transmission of tuberculosis in the Tactile Perception: Oral test. Tuberculosis was declared an epidemic in the province (Western Cape Government, 2021), and the current provincial prevalence rate is 0.75%. Tuberculosis is spread through the air (Western Cape Government, 2021), not through shapes placed in the mouth in this test. However, the perception of infection risk could impact the willingness of participants to take part. On the other hand, the Tactile Perception: Oral test results may provide important information on oral sensitivities to help in understanding the child’s SR vulnerabilities and potential relationship to fussy eating (Parham et al., 2021). Fussy eating is a common occupational outcome of SR difficulties. After weighing up these factors, the PI decided to retain this test.

It was difficult to compare these cognitive interview findings with other SR test adaptation studies, which reported minimal information on results of cognitive interviews (Hadgu and Zeleke, 2017; Lai et al., 2011; Román-Oyola and Reynolds, 2010; Shahbazi et al., 2021; Su and Parham, 2002). In these studies, feedback on contextual appropriateness was frequently elicited from professionals and not caregivers (Hadgu and Zeleke, 2017; Shahbazi et al., 2021). Although challenges facing caregivers were reported, the numbers of caregivers involved were reported in only one study (Román-Oyola and Reynolds, 2010), making this information less valuable and less interpretable.

### Item content validity ratings

The last aspect of content validity to be evaluated involved item and subscale content validity rating and the calculation of the percentage of agreement (Thorn and Deitz, 1989). The item and subscale content validity ratings for the performance-based assessment component demonstrated strong content validity. Ratings varied for the caregiver questionnaire items. The following section will focus only on discussion of the item content validity ratings of the SPM-2 Child questionnaire, as there was considerable overlap between the SPM-2 Child and SPM-2 Preschool items.

The *vision* subscale measures a variety of aspects of visual functioning and demonstrated insufficient content validity as a measure of visual reactivity. Five of the 10 items were judged to evaluate visual perception, distractibility, or oculo-motor skills rather than visual SR. This aligned with findings of another study (Dugas et al., 2018) and with the test developers of the SPM-2 (Parham et al., 2021). The five items that measured other constructs were excluded. This resulted in sufficient content validity for the vision subscale, as all remaining items in the subscale measured the construct.

*Hearing* and *touch* items and subscales had sufficient item content validity ratings and demonstrated the highest ratings of the six sensory subscales. One tactile item did not measure the construct of SR: ‘[This child] has trouble finding things in a pocket, bag, or backpack without looking’. This item was interpreted by occupational therapists in the study and by the SPM-2 test developers to evaluate tactile perception and not tactile reactivity (Parham et al., 2021). However, all auditory and tactile items were retained as more than 80% of the items in these subscales measured the construct of reactivity. Sensitivities in the auditory and tactile systems (which Ayres termed auditory defensiveness and tactile defensiveness, respectively) were the first types of SR reported in the literature (Ayres, 1972; Knickerbocker, 1980). Familiarity of occupational therapists with these behaviours may have made it easier to generate SR items for these systems.

The *taste and smell* sensory systems were grouped together in one subscale and demonstrated sufficient item content validity. One item with insufficient content validity stated: ‘[This child] fails to distinguish or indicate preference among flavours such as sweet/sour/bitter’. This item was judged by occupational therapists in the study to evaluate gustatory perception rather than reactivity. This contradicted the interpretation by the SPM-2 test developers who considered that this item primarily represented gustatory hypo-reactivity. The test developers did, however, state that while this interpretation primarily fitted the behaviour of the item, there may be other interpretations (Parham et al., 2021). All olfactory and gustatory subscale items were retained in the final version. The olfactory and gustatory systems play key roles in eating (Dugas et al., 2018; Parham et al., 2021). The strong validity scores for these two systems may be due to the familiarity of occupational therapists who see many cases of children with fussy eating, making it easier to generate test items relevant to SR.

The *body awareness* subscale refers to proprioceptive sensory input (Parham et al., 2021). Content validity ratings for these items was low. Occupational therapists in the study identified only 2 of the 10 items as measuring this construct. However, these two items reflected sensory seeking behaviours, which was excluded in the definition of SR used in this study. The SPM-2 test developers also interpreted these two items as reflecting sensory-seeking behaviours (Parham et al., 2021). The remaining items were interpreted by the occupational therapists and the SPM-2 test developers as indicating perception of proprioceptive input (Parham et al., 2021). Therefore, none of these subscale items measured the construct of SR, and the subscale was removed in its entirety.

Although the validity rating for the proprioceptive subscale did not indicate any proprioceptive reactivity items, other assessments frequently do contain items purported to test proprioceptive reactivity. It would appear that the general assumption has been that SR can be present in any sensory system (Sutthachai et al., 2022). However, this may not be the case. The developers of the Thai Sensory Processing Assessment noted difficulties interpreting their proprioceptive reactivity findings (Sutthachai et al., 2022). They reported that proprioception items had the lowest internal consistency ratings, a measurement property closely linked to content validity (Sutthachai et al., 2022; Terwee et al., 2018b). They recommended triangulation of the test results with other data (Sutthachai et al., 2022). In addition, in the literature, proprioception is often grouped with vestibular functions for both reactivity and perception, as in ‘vestibular-proprioceptive difficulties’. This may mask the fact that the reactivity difficulties may arise from the vestibular but not the proprioceptive system or may reflect proprioceptive perception rather than reactivity. Furthermore, neither Ayres’ writings nor her factor analyses report on proprioceptive SR (Ayres, 1972). This study’s findings regarding proprioceptive reactivity support current anecdotal thinking from experts in the field suggesting that proprioceptive reactivity is either rare or does not exist (Parham et al., 2021). However, this view has not been confirmed by research.

The *balance and motion* subscale reflects vestibular function (Parham et al., 2021). Insufficient item content validity ratings were demonstrated in this subscale, with six items judged to reflect balance, motor control and co-ordination. This was in line with another study (Dugas et al., 2018) and with the interpretation of the SPM-2 test developers (Parham et al., 2021). These six items were removed, leaving four items in the subscale. The percentage of agreement for items in this subscale reflecting vestibular reactivity was now calculated at 75%, below the 80% threshold set in this study for sufficient subscale content validity. However, the PI thought that the benefit in maintaining the vestibular subscale outweighed the five-percentage point loss in sufficiency rating. This was due to the importance of the vestibular system in Ayres Sensory Integration^®^ and in the construct of SR (Ayres, 1972), one of the earlier sensory systems in which Ayres identified sensory hyper-reactivity. She referred to this vestibular hyper-reactivity as gravitational insecurity (Ayres, 1972).

There were a number of *limitations* in this study. The first was the imbalance in the power relationship between the occupational therapy testers and the participants due to the language challenges. While steps were taken to address this, it is possible that this may still have affected some participants, leading to bias in their responses. A second limitation was the use of an English-language caregiver questionnaire. This likely resulted in the insufficient content validity finding for comprehensibility due to numerous challenges reported by some caregivers whose home language was not English. Many of these challenges may have been addressed if these caregivers had been provided with a translator who read the questionnaire in their home language.

A third limitation was that the PI did not formally test caregivers’ reading ability prior to inclusion in the feasibility study, but rather asked for the caregiver’s highest educational level. A grade-five reading level was the criteria set in the study, being the guideline recommended in the literature (Beaton et al., 2007). However, a grade five or higher educational level may not be an accurate indication of the caregiver’s ability to read with understanding at the recommended level.

A fourth limitation was that this study only focused on the SR component of sensory integration. There are however four categories included in the theoretical framework of Ayres Sensory Integration (Mailloux et al., 2020) on which this study was based. All aspects of sensory integration need to be evaluated. It is hoped that other researchers will perform content validity studies on assessment of the other three aspects of sensory integration.

A fifth limitation was the use of the EASI Research Version. Ceiling and floor levels had not been determined, nor the number of failed items to warrant termination of the test (Mailloux et al., 2020). The test was therefore lengthy, with some children struggling to remain motivated and focused. To address this, the PI requested that testers use their clinical judgement to determine when the child was unlikely to be able to make any further correct responses. The final version of the EASI is likely to have some item reduction, ceiling and floor levels, and discontinuation criteria. These will all result in a shortened test administration time.

Several *recommendations* arose out of this study. The translation process from English language to an indigenous language should involve a back translation process to ensure that translation is accurate. Reading and comprehension in English is challenging for many of the people of the Western Cape province, as English is their second or third language. Translation into Afrikaans and isiXhosa, the other two official languages of the Western Cape province, is recommended. Translation into other commonly used languages in South Africa, such as isiZulu, is also recommended.

Translation should be followed by normative data collection for the development of South African norms and the evaluation of other psychometric properties of reliability and validity. These steps require a larger sample that is representative of the South African population.

## Conclusion

When a test is used in a context different from where it was originally developed, it is recommended that cultural adaptation and translation as well as evaluation of content validity, at least, be conducted. This study developed a novel methodology for evaluating content validity. This article describes the methodology as it pertains to the evaluation of content validity of two SR assessments that were adapted for use in the Western Cape province of South Africa. The methodology developed for this study provides a rich, multifaceted format for evaluating content validity and can serve as a model for others who wish to evaluate content validity in the development of assessments or test adaptations.

**Key findings**

- A comprehensive methodology for evaluating content validity was developed.
- Thresholds for determining sufficient content validity were set.
- Content validity must be re-evaluated when tests are used in new contexts.

**What the study has added**

A novel methodology for evaluating content validity was developed and its use demonstrated on two sensory reactivity assessments adapted for the South African context.
